# Current Challenges in the Treatment of Invasive Aspergillosis in Geriatric Patients

**DOI:** 10.3390/jof11070480

**Published:** 2025-06-25

**Authors:** Sara Fueyo Álvarez, Elena Valle Calonge, Julieth Andrea Caballero Velasquez, Alba Magaly Revelo Rueda, Pablo Enrique Solla Suarez, Eva María López Álvarez, Mercedes Rodriguez Perez, María Teresa Peláez García de la Rasilla

**Affiliations:** 1 Hospital Monte Naranco, 33012 Oviedo, Spain; elena.valle@sespa.es (E.V.C.);; 2 Hospital Central de Asturias, 33011 Oviedo, Spain; 3 Instituto de Investigaciones Sanitarias del Principado de Asturias (ISPA), 33011 Oviedo, Spain

**Keywords:** invasive aspergillosis, geriatrics, mortality

## Abstract

**Background:** Invasive aspergillosis (IA) is a severe fungal infection increasingly affecting elderly patients with chronic respiratory diseases and prolonged corticosteroid use. **Methods:** We evaluated clinical, biochemical, and fungal biomarkers in 45 patients over 80 years diagnosed with IA and hospitalized in a Spanish Acute Geriatric Unit. Patients received either voriconazole or isavuconazole. Mortality rates and associated risk factors were analyzed. **Results:** Overall mortality was 35.61%. Significant mortality risk factors included leukocytosis (*p* = 0.0371), neutrophilia (*p* = 0.0144), and lymphopenia (*p* = 0.0274). Deceased patients had longer hospital stays (26.6 vs. 16.8 days; *p* = 0.00353). Voriconazole treatment was associated with higher 30-day mortality (61.5% vs. 19.2%; *p* = 0.0001) and a higher incidence of adverse effects (60% vs. 5%; *p* = 0.0003) compared to isavuconazole. Voriconazole also showed greater pharmacokinetic variability, with 76.9% of cases outside the therapeutic range. **Conclusions:** Voriconazole may not be optimal for IA treatment in patients over 80 years. Isavuconazole demonstrated a more favorable safety and efficacy profile. Personalized therapeutic strategies and a multidisciplinary approach are essential to improve clinical outcomes and quality of life in this vulnerable population.

## 1. Introduction

Invasive aspergillosis (IA) is a fungal infection associated with high morbidity and mortality, primarily caused by *Aspergillus fumigatus* and, to a lesser extent, by other species within the genus.

Approximately 6.5 million people are affected annually by invasive fungal infections and chronic pulmonary aspergillosis [1]. IA has become one of the most common causes of mortality among severely immunocompromised patients. In 2020, it was estimated that IA accounted for 4.5% of mortality in leukemia patients and 2.7% in lung cancer patients [1]. IA has also been recognized as a serious complication of COVID-19 infection, with an incidence ranging from 18% to 39% and a mortality rate of up to 50% [2].

In a 2017 study of hospitalized patients, the incidence of IA was found to be 3.54 per 100,000 inhabitants/year in Spain and 2.32 per 100,000 inhabitants/year in the Principality of Asturias [3].

Traditionally considered a disease of severely immunocompromised patients, such as those undergoing transplants or cancer treatments, recent years have shown a rising incidence in elderly populations.

In patients older than 80 years, intrinsic aging of the immune system (immunosenescence), chronic comorbidities, and decreased physiological reserve increase vulnerability to opportunistic infections, making IA an increasingly relevant clinical challenge [4,5].

Aging entails structural and functional changes in multiple systems, notably the deterioration of the immune response. Immunosenescence not only reduces the capacity for an adequate inflammatory response but also impairs the effectiveness of defense mechanisms against fungal pathogens. Additionally, the coexistence of cardiovascular diseases, diabetes, chronic pulmonary diseases, and other disorders in elderly populations complicates diagnosis, as initial symptoms of IA can overlap with manifestations of these underlying conditions. Therefore, clinical suspicion and the use of specialized diagnostic tools are essential for the early detection of the infection.

*Aspergillus* infection is diagnostically challenging, requiring a high index of clinical suspicion. The “gold standard” diagnostic methods include histological studies, fungal culture growth, galactomannan (GM), 1,3 β-D-glucan assays, polymerase chain reaction (PCR) tests, and rapid immunochromatographic tests such as *Aspergillus* lateral flow device (LFD) and lateral flow assay (LFA) [6].

The diagnosis of IA in octogenarian and nonagenarian patients relies on integrating clinical, radiological, and microbiological criteria. High-resolution computed tomography (CT) has become the method of choice to identify suggestive radiological patterns, such as the “halo” or “cavity” signs, although its specificity can be limited by pulmonary comorbidities. Combining these diagnostic tools with thorough clinical evaluation is crucial for timely and accurate diagnosis in this patient cohort [7].

Regarding treatment, IA requires individualized therapeutic strategies. Triazole antifungals, such as voriconazole, constitute the first-line treatment, having demonstrated efficacy in multiple clinical studies. However, in patients over 80 years old, consideration must be given to potential drug interactions and pharmacokinetic changes due to reduced hepatic and renal function, which may amplify adverse effects. Monitoring plasma levels of voriconazole and closely tracking organ function is essential for dose adjustment and toxicity minimization [8]. Additionally, polypharmacy is common in these patients, necessitating a careful review of possible drug interactions, further complicating clinical management.

The European Public Assessment Report (EPAR) from the European Committee for Medicinal Products for Human Use evaluated these medications for marketing authorization, providing usage recommendations. Concerning elderly patients, the only note made in the summary of product characteristics is that, in men older than 65 years, the maximum concentration is 61% higher compared to patients aged 18–45 years, and the area under the curve—reflecting duration, average concentration, and bioavailability—is 81%. No data are available for women, nor on age-based dose adjustments; thus, the same side effects assumed for younger adults are anticipated [9].

The heterogeneous clinical presentation of IA in elderly populations underscores the need for adapted diagnostic and therapeutic guidelines. Recent studies highlight that IA may present atypically in older patients, with milder symptoms or mimicking exacerbations of pre-existing pulmonary diseases. This scenario requires clinicians to maintain a high index of suspicion and apply diagnostic algorithms incorporating imaging techniques, microbiological, histological, serological, and molecular testing. Prompt initiation of treatment has been associated with improved prognosis, emphasizing the importance of early detection and timely intervention [5].

The objectives of this study are to determine mortality rates and associated risk factors among elderly hospitalized patients diagnosed with IA, and to analyze whether the type of antifungal treatment influences 30-day mortality.

## 2. Materials and Methods

### 2.1. Study Design

An observational, analytical, multicenter, retrospective-prospective study was conducted at two hospitals, one specializing in infectious diseases and geriatric care. The study period spanned from 1 January 2018 to 13 January 2023. Patients were retrospectively included from 1 January 2018 to 30 June 2022 and prospectively from 1 November 2022 to 13 January 2023.

The retrospective study included patients aged over 80 hospitalized in departments of Geriatrics, Internal Medicine, Pneumonology, and Intensive Care. The prospective study included patients aged over 80 admitted to the Acute Care Unit of the Geriatric Service.

The diagnostic process and therapeutic strategies implemented in patients aged 80 years or older diagnosed with IA were evaluated, following updated criteria from the European Organization for Research and Treatment of Cancer/Mycoses Study Group (EORTC/MSG) [5] and FUNDICU [10].

### 2.2. Population and Inclusion Criteria

Patients of both sexes aged ≥80 years were included, presenting clinical, radiological, and microbiological findings consistent with IA. Inclusion criteria were:

- Age ≥80 years.
- Diagnosis of IA based on EORTC/MSG and/or FUNDICU criteria, including suggestive radiological findings (e.g., halo sign, cavitations, or nodular infiltrates) combined with positive microbiological results (culture, LFD/LFA, galactomannan, and/or *Aspergillus*-specific PCR).
- Availability of complete clinical information and a minimum follow-up of 90 days after initiation of antifungal treatment.

Patients with uncertain diagnoses, terminal comorbidities precluding objective clinical evaluation, or insufficient documentation in medical records were excluded.

### 2.3. Diagnostic Procedures

All patients underwent evaluation following a standardized diagnostic protocol, including:

1. Clinical Evaluation:

Detailed collection of medical history, assessment of respiratory and systemic symptoms, and comprehensive physical examination. Functional status and frailty assessment using validated scales (Barthel Index and Fried Frailty Scale) to determine physiological reserve and correlate with treatment response. Cognitive evaluation using validated scales such as Reisberg’s Global Deterioration Scale (GDS), determining cognitive stages from early deficits to severe symptoms.

2. Radiological Studies:

High-resolution computed tomography (HRCT) was performed in all cases to identify characteristic findings of infection, such as halo sign, cavitations, or nodular infiltrates. Chest radiographs complemented HRCT in situations where HRCT was not immediately available, following current diagnostic protocols suggested in the literature [11].

3. Microbiological and Laboratory Tests: The microbiological analysis methodology included:

Fungal cultures of respiratory samples using Sabouraud dextrose agar (BioMerieux, Mercy L’Etoile, France), supplemented with chloramphenicol (500 mg/L), were incubated at 30 °C for four weeks. Fungal identification performed by matrix-assisted laser desorption/ionization time-of-flight mass spectrometry (MALDI-TOF; Bruker, Bremen, Germany) per manufacturer’s guidelines.

Real-time quantitative PCR for *Aspergillus* species was performed as follows: DNA extraction and qPCR using the ELITe InGenius automated platform targeting the fungal 18S ribosomal DNA (r18S rDNA) region, and the human beta-globin gene as an internal standard. The number of fungal DNA copies expressed as copies/mL based on an 18S rDNA standard curve. Sputum samples were processed with 5% N-acetyl-L-cysteine (1:1 ratio) for liquefaction and inoculated undiluted in culture media within 24 h of collection.

The LFD for *Aspergillus* (AspLFD, OLM Diagnostics, Newcastle upon Tyne, UK) is an immunochromatographic test that detects gliotoxin from *Aspergillus* spp. in clinical samples. The test was read using a visual reader that provides a semi-quantitative assessment and eliminates subjectivity in result interpretation. Galactomannan (GM) test performed using Platelia^TM^ *Aspergillus* (Bio-Rad Laboratories, Madrid, Spain), with cut-off values ≥0.5 in serum, ≥1.0 in bronchoalveolar lavage (BAL), ≥4.0 in tracheal or bronchial aspirate, and ≥0.7 in sputum due to lack of standardization [12]. *Aspergillus* lateral flow assay (LFA) (IMMY LFA) [6] is an immunochromatographic diagnostic technique that detects galactomannan (GM), quantified using a lateral flow reader (LF Reader, IMMY, Norman, OK, USA) for standardized and accurate results.

General laboratory tests, including complete blood count, liver and renal function tests, and inflammatory markers, to assess patient baseline condition and identify alterations potentially influencing antifungal drug pharmacokinetics [8].

### 2.4. Therapeutic Procedures

Antifungal management was implemented according to international guidelines, adjusted to elderly population characteristics:

1. Antifungal Treatment:

Voriconazole was used as a first-line agent, administered at recommended doses adjusted for each patient’s hepatic and renal function. In cases of contraindication or intolerance to voriconazole, isavuconazole was considered an alternative according to recent recommendations [4,11].

Therapeutic drug monitoring via plasma voriconazole levels was employed to optimize treatment and minimize toxicity.

2. Follow-up and Therapeutic Response Assessment:

Patients were systematically reevaluated at 7, 14, and 30 days post-initiation of antifungal therapy. Repeat radiological studies (preferably TC) and laboratory tests were performed to evaluate clinical evolution, treatment response, and early detection of adverse effects. Given the high polypharmacy rate in this cohort, potential drug interactions were documented and analyzed, making necessary adjustments in antifungal therapy and concomitant medication.

### 2.5. Data Collection and Management

Demographic variables (age and sex), geriatric variables (functional and cognitive status), care-related factors (length of hospital stay), and clinical variables (diabetes, COPD with GOLD classification, structural lung abnormalities, home oxygen therapy, steroid use ≥48 h in previous 3 months, antibiotic therapy in previous 3 months, prior immunosuppressant use, antifungal treatment) were recorded.

Additionally, leukocyte counts, neutrophils (percentage and absolute count), lymphocytes (percentage and absolute count), albumin, and interleukin-6 (IL-6) levels were collected. Mortality data during hospitalization and at 30 days post-discharge were also recorded.

Information was collected through a standardized electronic form, including demographic variables, clinical history, comorbidities, radiological findings, microbiological test results, antifungal treatment details (dose, duration, adjustments), and clinical outcomes (treatment response, complications, mortality). Data was anonymized and securely stored according to current personal data protection regulations.

### 2.6. Statistical Analysis

Data analysis was performed using SPSS version 25.0 (IBM Corp., Armonk, NY, USA). A descriptive analysis of variables was conducted: Categorical variables expressed as frequencies and percentages, and continuous variables summarized as means and standard deviations or medians and interquartile ranges, assessed by the Kolmogorov–Smirnov test.

Chi-square or Fisher’s exact test was used for categorical variables, and Student’s *t*-test or Mann–Whitney U test for continuous variables. A *p*-value < 0.05 was considered statistically significant. Multivariate logistic regression analysis identified independent factors associated with therapeutic response and mortality.

Statistical analysis was also conducted using R software (version 4.2.0.), employing the non-parametric Kruskal–Wallis test for quantitative variables and the Chi-square test for qualitative variables.

### 2.7. Ethical Considerations

The study was approved by the Ethics Committee for Research of [Hospital Universitario Central de Asturias] (protocol number: 538-2022). Informed consent was obtained from patients or legal representatives in the prospective phase, ensuring compliance with the principles of the Declaration of Helsinki and national legislation regarding clinical research.

## 3. Results

### 3.1. Patient Selection and Demographics

**Figure 1:**
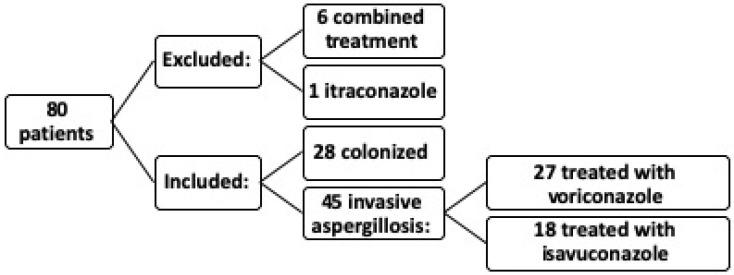
Patients included and excluded from the study.

In the study, out of a total of 80 patients, 6 patients were excluded due to receiving combined treatment with voriconazole and the potential interaction between different medications, which could introduce confounding bias. Additionally, one patient treated with itraconazole was excluded because a single patient was insufficient to constitute a significant sample. Ultimately, the study was conducted on 73 patients (Figure 1).

Of the 73 patients included, 45 were diagnosed with IA, with a mean age of 88 years; 56.2% of these were male. Of these 45 IA patients, 27 were treated with voriconazole and 18 with isavuconazole, with an overall mortality of 35.61%. The remaining 28 patients were considered colonized according to EORTC and FUNDICU criteria.

### 3.2. Comparison Between Deceased and Surviving Patients with Invasive Aspergillosis

Table 1 and Table 2 detail comparative data between deceased patients with IA (n = 26) and those who survived (n = 47). Among patients with IA, both deceased and survivors, there were no significant differences in age, with a mean age of 88.8 years in the overall population. There were also no statistically significant differences regarding sex; although there was a higher proportion of males in the deceased group (69.2% vs. 48.9%), this difference was not statistically significant.

Regarding functional status, no statistically significant differences were observed. However, from a geriatric perspective, it is important to highlight that both deceased and surviving patients were mostly functionally independent or presented with mild to moderate dependency. The Barthel Index was greater than 60 in 72.3% of survivors and 65% of deceased patients. No statistically significant differences were found in the level of cognitive impairment between the two groups.

In terms of comorbidities, such as chronic obstructive pulmonary disease (COPD), structural lung alterations, diabetes, and predisposing treatments (home oxygen therapy, corticosteroids, immunosuppressants, and antibiotics in the previous three months), no significant differences were identified between deceased and surviving IA patients.

Comparative analysis revealed significant differences in several clinical parameters and biomarkers, identifying risk factors associated with mortality in this elderly population.

Specifically, two statistically significant clinical differences between deceased and surviving IA patients were observed. Length of hospital stay, with deceased patients having a significantly longer hospitalization period (26.6 days for deceased vs. 16.8 days for survivors; *p* = 0.00353). Mortality by antifungal treatment: Treatment with voriconazole was associated with higher mortality, while isavuconazole demonstrated better survival outcomes. Thirty-day mortality was significantly lower in patients treated with isavuconazole (19.2%) compared to those exclusively treated with voriconazole, who had a 30-day mortality rate of 61.5% (*p* = 0.00426).

Three biochemical biomarkers showed significant differences between deceased and surviving IA patients: Leukocyte count, with deceased patients presenting significantly higher leukocytosis (14,000/mm^3^) compared to survivors (9720/mm^3^; *p* = 0.0371); Absolute neutrophil count, with higher neutrophil levels observed among deceased patients (12,100/mm^3^) compared to survivors (7700/mm^3^), suggesting greater inflammatory response (*p* = 0.0144); Lymphocyte percentage, with deceased patients presenting significantly more pronounced lymphopenia (4.46%) compared to survivors (10.2%; *p* = 0.0274), possibly indicating a poorer immune response.

No significant differences were observed between deceased and surviving IA patients regarding fungal biomarkers such as β-glucan levels (*p* = 0.375), galactomannan (GM) (*p* = 0.551), *Aspergillus* PCR (*p* = 0.725), and positive cultures (*p* = 0.405). Similarly, no significant differences were found in biochemical markers such as albumin (*p* = 0.18) or IL-6 (*p* = 0.538). Notably, albumin levels were lower among deceased patients (30.4 vs. 33.4 g/L), although this did not reach statistical significance.

In summary, deceased patients had a significantly longer hospital stay, leukocytosis, neutrophilia, and lymphopenia, suggesting an intense inflammatory response and possible immune dysfunction. Moreover, treatment with voriconazole was associated with higher mortality compared to isavuconazole.

### 3.3. Comparison of Antifungal Treatments

Based on these results, a comparative analysis was performed between IA patients treated with voriconazole versus isavuconazole (Table 3 and Table 4), in the context of IA within the geriatric population, revealing significant differences across several clinical parameters, biochemical biomarkers, and fungal biomarkers.

The four statistically significant clinical differences were (Table 3): Length of hospital stay: A significant reduction in hospitalization duration was observed in patients treated with isavuconazole (20.2 days) compared to those treated with voriconazole (28.6 days; *p* = 0.0046). In-hospital mortality: Initial mortality during hospitalization was higher in the voriconazole group (29.6%) compared to the isavuconazole group (22.2%; *p* < 0.0001). 30-day mortality: A significant difference in 30-day mortality was noted, being substantially lower in the isavuconazole-treated group (5.6%) compared to the voriconazole group (40.7%; *p* < 0.0001). Use of immunosuppressants: Patients treated with voriconazole (1.11) received immunosuppressive medications more frequently than those treated with isavuconazole (1.00; *p* = 0.0017).

Importantly, no statistically significant differences were observed in age, sex, cognitive impairment, or functional status between patients treated with voriconazole and those treated with isavuconazole, thus not influencing the outcomes. Moreover, no significant differences were found in comorbidities analyzed, such as chronic obstructive pulmonary disease (COPD), structural pulmonary alterations, diabetes, or predisposing treatments including home oxygen therapy, corticosteroids, and antibiotic use within the previous three months.

Regarding statistically significant differences in biochemical and fungal biomarkers between patients treated with voriconazole versus isavuconazole (Table 4), the following were identified: β-D-glucan: Higher β-D-glucan levels were identified in the isavuconazole-treated group (1.00 pg/mL) compared to the voriconazole group (0.741 pg/mL; *p* = 0.0169). Galactomannan (GM): Higher GM levels were observed in patients treated with isavuconazole (3.61) versus voriconazole (2.28; *p* = 0.0153). *Aspergillus* PCR: Patients treated with voriconazole had significantly higher PCR levels (84,400) compared to those receiving isavuconazole (4990; *p* = 0.0192). Neutrophil percentage: A highly significant difference in neutrophil counts was identified, with a higher neutrophil percentage among patients treated with isavuconazole (82.2%) versus voriconazole (32.1%; *p* < 0.0001). Lymphocyte percentage: A significant reduction in lymphocyte percentage was noted among patients treated with voriconazole (2.92%) compared to isavuconazole (14.8%; *p* < 0.0001). Total lymphocyte count: Significantly lower absolute lymphocyte counts were observed in the voriconazole group (936/mm^3^) compared to the isavuconazole group (1630/mm^3^; *p* = 0.0104). Albumin levels: Significantly lower albumin levels were noted in the voriconazole group (29.7 g/L) compared to patients treated with isavuconazole (34.2 g/L; *p* = 0.0015).

No significant differences were found between IA patients treated with voriconazole and those treated with isavuconazole regarding fungal biomarkers such as LFD (*p* = 0.968), LFA (*p* = 0.093), and positive cultures (*p* = 0.113), nor in biochemical markers including absolute neutrophil count (*p* = 0.090) and IL-6 levels (*p* = 0.0755).

In conclusion, patients treated with voriconazole experienced higher mortality rates both during hospitalization and at 30 days, as well as a longer duration of hospital stay. Conversely, isavuconazole demonstrated a better safety profile, with lower mortality rates, shorter hospitalization time, and improved biochemical parameters (higher albumin levels, higher percentages of lymphocytes and neutrophils, and higher absolute lymphocyte counts).

These findings support reconsidering the use of voriconazole in geriatric patients with IA, favoring the use of isavuconazole due to its lower toxicity and better clinical outcomes.

### 3.4. Adverse Effects and Pharmacokinetics

**Figure 2:**
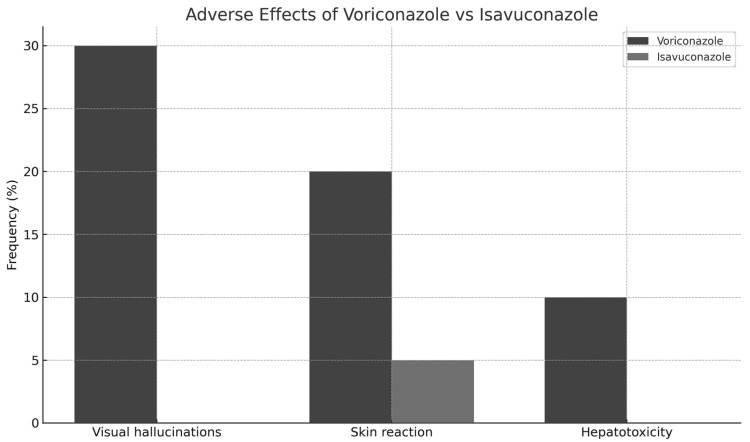
Adverse effects in patients according to treatment: Voriconazole vs. isavuconazole.

Finally, regarding the side effects of both antifungal agents (Figure 2), it was observed that 60% of patients treated with voriconazole experienced significantly greater adverse effects (*p* = 0.0003), compared to only 5% of those treated with isavuconazole. The most prominent adverse effects were:

- Visual hallucinations: Reported in 30% of patients receiving voriconazole, versus 0% in the isavuconazole group.
- Skin rash: Observed in 20% of patients treated with voriconazole compared to 5% in the isavuconazole group.
- Hepatic toxicity: Detected in 10% of patients treated with voriconazole, absent in the isavuconazole-treated group.

About of the patients treated with voriconazole, 76.95% were within the therapeutic range, which is 1–5.5 μg/mL. However, 23.05% of patients had voriconazole levels outside the therapeutic range, specifically, 7.67% had plasma levels above the range. The mean patient concentration was 3.9 μg/mL (range: 0.87–5.72 μg/mL).

Isavuconazole exhibits predictable and linear pharmacokinetics, with low interindividual variability compared to voriconazole. According to the EMA’s (European Medicines Agency) EPAR (European Public Assessment Report), systematic monitoring of plasma levels is not considered necessary in routine clinical practice [9]. The efficacy and safety profile of the dose administered to our patients was validated in pivotal studies such as SECURE or VITAL, with no need for monitoring [13].

It is noteworthy that all *Aspergillus* isolates from patients with IA included in the study were susceptible to both voriconazole and isavuconazole.

## 4. Discussion

IA in elderly patients, especially those over 80 years of age, represents a significant and underestimated clinical challenge in the fields of infectious diseases and geriatrics. Immunosenescence, along with epithelial barrier dysfunction and the presence of multiple comorbidities, predisposes this population to opportunistic fungal infections, including IA [11]. Furthermore, the clinical presentation in these patients may be atypical and less predictable, making early diagnosis and the initiation of timely antifungal treatment difficult.

This study provides key evidence on IA-associated mortality in geriatric patients, highlighting the impact of antifungal treatment on clinical outcomes. Although previous studies have analyzed IA in older adults, most have focused on patients over 65 years of age without distinguishing advanced age subgroups. This study is the first to evaluate a cohort of patients ≥80 years of age, providing valuable information on the management of a highly vulnerable population. The findings of this study indicate that IA in elderly patients has a 35.6% mortality rate, significantly associated with hematological alterations such as leukocytosis, neutrophilia, and lymphopenia. These biomarkers have been identified as prognostic factors for mortality, highlighting the need for more precise diagnostic and therapeutic strategies in this population [14]. Immunosenescence, characterized by a decrease in the number and function of lymphocytes, affects both the innate and adaptive immune responses, reducing the body’s ability to control the fungal burden and responding less effectively to antifungal treatment [15,16]. Immunosenescence has been shown to impact the inflammatory response in invasive fungal infections, which could explain the high mortality observed in elderly patients with IA [17]. In this sense, comprehensive immunological evaluation at the start of treatment emerges as a key factor to predict adverse outcomes and optimize therapeutic management.

Previous research has indicated that therapeutic failure with voriconazole is associated with hepatotoxicity and acquired resistance to azoles, even in the presence of adequate plasma concentrations [14]. However, few studies have evaluated the influence of immune status on IA mortality. In this study, statistically significant differences in patient survival were identified based on their leukocytosis, neutrophilia, and lymphopenia levels, which compromise the host immune response and reduce its ability to control the infection [15]. Several studies have reported that mortality from invasive pulmonary aspergillosis in older adults is extremely high, reaching up to 82.9% in critically ill patients, compared with 77% in the general population [15,16]. Additionally, bacterial coinfection, particularly with Gram-negative pathogens, has been identified as a key determinant of mortality in these patients. Lymphopenia has been identified as a significant predictor of secondary infections, highlighting the need to monitor lymphocyte counts and implement preventive strategies to minimize the impact of coinfections in patients with IA [16,18].

The immunological status of patients plays a critical role in the outcome of IA. This study identified a significant association between leukocytosis, neutrophilia, and lymphopenia with worse clinical outcomes, highlighting the need to assess immune status at admission as a key prognostic factor [16,19]. The results obtained in this study emphasize the high vulnerability of patients ≥80 years of age with IA, justifying the implementation of early detection protocols and the intensification of surveillance in geriatric and intensive care units. The identification of hematological biomarkers such as leukocytosis, neutrophilia, and lymphopenia may facilitate better risk stratification and guide therapeutic decision-making more accurately. Evidence suggests that a comprehensive therapeutic approach, combining antifungal treatment to combat the infection, along with immune modulation strategies to improve the patient’s immunocompetence and control coinfections, could represent a promising avenue to improve clinical outcomes in this highly vulnerable population. One of the main scientific contributions of this study is the key evidence on mortality associated with IA in geriatric patients, highlighting the impact of antifungal treatment on clinical outcomes. A significant difference in patient outcomes was observed depending on the antifungal used, showing that the exclusive use of voriconazole was associated with higher mortality and a longer hospital stay compared to isavuconazole. These findings are consistent with previous studies suggesting a lower effectiveness of voriconazole in older adults, attributed to its complex pharmacokinetic profile and potential liver toxicity [20].

In this cohort, patients treated with voriconazole had a 30-day mortality rate of 61.5%, in addition to a high incidence of adverse effects (60%), in contrast to the isavuconazole group, whose mortality was 19.2% and adverse effects were limited to 5%. This significant difference raises the need to reconsider current therapeutic guidelines for this high-risk population. Voriconazole pharmacokinetics in elderly patients showed high variability, with plasma levels outside the therapeutic range in 23.05% of cases, which could contribute to its greater toxicity and mortality [5,21].

The available evidence on antifungal treatment in elderly patients is limited, and although some studies have supported the safety and efficacy of voriconazole in older adults [22,23], others have pointed to its association with higher mortality in this population [24,25]. The inferiority of voriconazole in geriatric patients may be explained by the progressive deterioration of liver and kidney function with age, which affects its metabolism and can result in suboptimal or toxic plasma concentrations [26].

In contrast, isavuconazole presented a more stable pharmacokinetic profile and more predictable bioavailability, with fewer drug interactions and reduced liver toxicity, making it a safer alternative for elderly patients taking multiple medications [27]. Our results are consistent with the SECURE study (NCT00412893) [13], which demonstrated the superiority of isavuconazole over voriconazole in terms of safety and efficacy, although the mean age in that study was 70 years, while the present study evaluated a population with a mean age of 88 years [11,28].

In this context, isavuconazole represents a more appropriate therapeutic option, emphasizing the need to update clinical guidelines to reflect emerging evidence on the treatment of IA in older adults.

Another important consideration is that the length of hospitalization is a determining factor in the clinical course of elderly patients with IA, not only due to the impact on individual prognosis but also due to the implications for the use of healthcare resources and hospital costs. In this vulnerable population, a prolonged hospital stay significantly increases the risk of nosocomial complications, including secondary infections, functional decline, and malnutrition, which worsen morbidity and may contribute to an unfavorable clinical outcome [29].

This finding is consistent with previous studies suggesting that the variable pharmacokinetics of voriconazole in older adults, along with its potential hepatic and neurological toxicity, may increase the need for close monitoring and dose adjustments, potentially prolonging hospitalization [19].

In addition to the risk of nosocomial infections, prolonged hospitalization in elderly patients is associated with accelerated functional decline and a higher prevalence of geriatric syndromes, such as sarcopenia and malnutrition, further compromising recovery and quality of life after hospital discharge [30]. Optimization of antifungal treatment by selecting agents with more favorable safety profiles, such as isavuconazole, and implementing therapeutic monitoring strategies, could contribute to reducing the length of hospital stay and minimizing associated complications, thus improving clinical outcomes in this high-risk population [11].

Finally, this study is particularly relevant in the current context due to the progressive aging of the global population and the increase in opportunistic infectious diseases in this age group [22]. With increasing life expectancy and the widespread use of immunosuppressants, IA has become a growing concern in the fields of infectious diseases and geriatrics. Therefore, the need for effective and safe therapeutic strategies is more urgent than ever.

From a clinical perspective, this study contributes relevant data to the scientific literature, as it focuses on an age group that has been underrepresented in previous studies. Most previous studies on IA have included patients over 65 years of age, but without differentiating between elderly subgroups [4]. The exclusive inclusion of patients over 80 years of age in this study provides crucial information for the management of this highly vulnerable subgroup and allows for the identification of specific challenges in antifungal treatment for these patients.

Regarding its strengths, we highlight the specific focus on an understudied age group, those over 80 years of age, and its detailed evaluation of the impact of antifungal treatment in this vulnerable and demographically growing population. Despite this, the study has certain limitations, including the choice of treatment at the discretion of the treating physician, which could be a bias. Furthermore, patients treated with antifungal combinations had to be excluded from the analysis due to the limited sample size for this type of treatment.

Future studies could focus on validating these findings through randomized clinical trials with larger sample sizes. It would also be interesting to include a more specific assessment of immunosenescence biomarkers to allow for more precise risk stratification in this population.

## 5. Conclusions

Our findings confirm the high mortality associated with invasive aspergillosis in elderly patients and suggest that the choice of antifungal treatment is crucial to improving clinical outcomes. The apparent superiority of isavuconazole over voriconazole in terms of safety and efficacy, along with the influence of immunological status at admission, opens new lines of research aimed at personalizing therapy in this vulnerable population.

## Figures and Tables

**Table 1:** Clinical characteristics of deceased and non-deceased patients with invasive aspergillosis.

	Total N = 73	Alive (N = 47)	Dead (N = 26)	*p*-Value
Age-Mean (SD)	88.8 (3.65)	88.6 (3.89)	89.4 (3.19)	0.48
Sex				
Men	41 (56.2%)	23 (48.9%)	18 (69.2%)	0.154
Women	32 (43.8%)	24 (51.1%)	8 (30.8%)	
Functional situation by groups				
Group 1 (BI de 0 a 60)	20.3 (14.3)	13 (27.7%)	10 (38.5%)	0.491
Group 2 (BI 60 a 100)	15.0 (47.0)	34 (72.3%)	16 (61.5%)	
Functional situation-Mean (SD)	69.9 (30.8)	72.6 (30.5)	65.0 (31.2)	0.179
Cognitive impairment				
No	61 (83.6%)	41 (87.2%)	20 (76.9%)	0.419
Yes	12 (16.4%)	6 (12.8%)	6 (23.1%)	
Admission time Mean (SD)	20.3 (14.3)	16.8 (12.5)	26.6 (15.3)	0.00353
Diabetes				
No	46 (63.0%)	30 (63.8%)	16 (61.5%)	1
Yes	27 (37.0%)	17 (36.2%)	10 (38.5%)	
COPD				
No	47 (64.4%)	33 (70.2%)	14 (53.8%)	0.253
Yes	26 (35.6%)	14 (29.8%)	12 (46.2%)	
Structural lung alterations				
No	38 (52.1%)	27 (57.4%)	11 (42.3%)	0.361
Yes	35 (47.9%)	20 (42.6%)	15 (57.7%)	
Oxygen therapy				
No	61 (83.6%)	40 (85.1%)	21 (80.8%)	0.882
Yes	12 (16.4%)	7 (14.9%)	5 (19.2%)	
Corticosteroids				
No	40 (54.8%)	27 (57.4%)	13 (50.0%)	0.714
Yes	33 (45.2%)	20 (42.6%)	13 (50.0%)	
Antibiotics in the last 3 months-Mean (SD)	1.42 (0.498)	1.38 (0.491)	1.50 (0.510)	0.336
Use of immunosuppressants-Mean (SD)	1.08 (0.277)	1.06 (0.247)	1.12 (0.326)	0.446
Treatment				
Isavucanazole	18 (24.7%)	13 (27.7%)	5 (19.2%)	0.00426
Voriconazole	27 (37.0%)	11 (23.4%)	16 (61.5%)	

**Table 2:** Biochemical and fungal biomarker (upon admission and 48 h later, respectively) data from deceased and non-deceased patients with IA.

	Total N = 73	Alive (N = 47)	Dead (N = 26)	*p*-Value
β-D-glucan-Mean (SD)	0.863 (0.673)	0.809 (0.647)	0.962 (0.720)	0.375
LFD-Mean (SD)	1.27 (0.961)	1.36 (0.942)	1.12 (0.993)	0.29
LFA-Mean (SD)	1.39 (2.89)	1.13 (1.67)	1.86 (4.29)	0.472
GM-Mean (SD)	2.43 (2.49)	2.49 (2.44)	2.33 (2.62)	0.551
PCR-Mean (SD)	89,300 (458,000)	113,000 (557,000)	45,500 (176,000)	0.725
Culture results-Mean (SD)	3.27 (5.18)	2.70 (4.45)	4.31 (6.26)	0.22
Leukocytes- Mean (SD)	11,300 (7850)	9720 (4320)	14,000 (11,400)	0.0371
Total neutrophils-Mean (SD)	9260 (6790)	7700 (3570)	12,100 (9820)	0.0144
Lymphocytes-Mean (SD)	8.17 (11.6)	10.2 (13.7)	4.46 (4.16)	0.0274
Albumin-Mean (SD)	32.3 (6.35)	33.4 (5.60)	30.4 (7.30)	0.18
IL6-Mean (SD)	67.8 (307)	88.8 (387)	33.0 (60.0)	0.538

**Table 3:** Clinical characteristics of patients with IA according to treatment.

	Voriconazole (N = 27)	Isavucanazole (N = 18)	*p*-Value
Age-Mean (SD)	88.0 (3.88)	88.9 (2.22)	0.295
Sex			
Men	21 (77.8%)	11 (61.1%)	0.383
Women	6 (22.2%)	7 (38.9%)	
Group 1 (BI de 0 a 60)	7 (25.9%)	4 (22.2%)	1.00
Group 2 (BI 60 a 100)	20 (74.1%)	14 (77.8%)	
Functional situation-Mean (SD)	71.9 (27.1)	74.2 (35.8)	0.830
Cognitive impairment			
No	24 (88.9%)	15 (83.3%)	0.929
Yes	3 (11.1%)	3 (16.7%)	
Admission time-Mean (SD)	28.6 (17.8)	20.2 (10.3)	0.0046
Diabetes			
No	16 (59.3%)	11 (61.1%)	1.00
Yes	11 (40.7%)	7 (38.9%)	
COPD			
No	16 (59.3%)	8 (44.4%)	0.502
Yes	11 (40.7%)	10 (55.6%)	
Structural lung alterations			
No	10 (37.0%)	11 (61.1%)	0.200
Yes	17 (63.0%)	7 (38.9%)	
Oxygen therapy			
No	21 (77.8%)	15 (83.3%)	0.939
Yes	6 (22.2%)	3 (16.7%)	
Corticosteroids			
No	15 (55.6%)	8 (44.4%)	0.670
Yes	12 (44.4%)	10 (55.6%)	
Antibiotics in the last 3 months-Mean (SD)	1.44 (0.506)	1.39 (0.502)	0.404
Use of immunosuppressants-Mean (SD)	1.11 (0.320)	1.00 (0)	0.0017
In-hospital mortality			
No	19 (70.4%)	14 (77.8%)	<0.0001
Yes	8 (29.6%)	4 (22.2%)	
30-day mortality			
No	11 (40.7%)	13 (72.2%)	<0.0001
Yes	8 (29.6%)	1 (5.6%)	

**Table 4:** Biochemical and fungal biomarker data (upon admission and 48 h later, respectively) from patients with IA according to treatment.

	Voriconazole (N = 27)	Isavucanazole (N = 18)	*p*-Value
β-D-glucan-Mean (SD)	0.741 (0.764)	1.00 (0.485)	0.0169
LFD-Mean (SD)	1.33 (0.961)	1.33 (1.03)	0.968
LFA-Mean (SD)	1.20 (2.47)	2.43 (4.38)	0.093
GM-Mean (SD)	2.28 (2.81)	3.61 (2.20)	0.0153
PCR-Mean (SD)	84,400 (257,000)	4990 (7780)	0.0192
Culture results-Mean (SD)	4.67 (6.06)	3.06 (5.48)	0.229
Leukocytes-Mean (SD)	12,600 (11,500)	10,300 (3770)	0.153
Neutrophils-Mean (SD)	32.1 (41.7)	82.2 (9.13)	<0.0001
Total neutrophils-Mean (SD)	10,600 (9910)	8240 (2570)	0.090
Lymphocytes-Mean (SD)	2.92 (4.65)	14.8 (18.2)	<0.0001
Total Lymphocytes-Mean (SD)	936 (608)	1630 (2250)	0.0104
Albumin-Mean (SD)	29.7 (7.15)	34.2 (5.17)	0.0015
IL6-Mean (SD)	138 (466)	19.6 (25.3)	0.0755

## Data Availability

The data presented in this study are available upon reasonable request from the corresponding author. The data are not publicly available due to institutional data protection policies concerning clinical records of geriatric patients.

## Abbreviations

The following abbreviations are used in this manuscript:

IA	Invasive aspergillosis
COPD	Chronic obstructive pulmonary disease
BAL	Bronchoalveolar lavage
HRCT	High-resolution computed tomography
PCR	Polymerase chain reaction
LFD	Lateral flow device
LFA	Lateral flow assay
GM	Galactomannan
SD	Standard deviation
BI	Barthel Index
GDS	Global Deterioration Scale
EORTC/MSG	European Organization for Research and Treatment of Cancer/Mycoses Study Group
FUNDICU	Fungal Infections in ICU study group
MALDI-TOF	Matrix-assisted laser desorption/ionization time-of-flight
IL-6	Interleukin-6
rDNA	Ribosomal DNA
SPSS	Statistical Package for the Social Sciences
IQR	Interquartile range
EPAR	European Public Assessment Report
EMA	European Medical Agency

## References

[1] Denning D.W. (2024). Global incidence and mortality of severe fungal disease. Lancet Infect. Dis..

[2] Jenks J.D., Nam H.H., Hoenigl M. (2021). Invasive aspergillosis in critically ill patients: Review of definitions and diagnostic approaches. Mycoses..

[3] González-García P., Alonso-Sardón M., López-Bernus A., Carbonell C., Romero-Alegría Á., Muro A., Galindo-Pérez I., Muñoz-Bellido J.L., Pardo-Lledias J., Belhassen-García M. (2021). Epidemiology of aspergillosis in hospitalised Spanish patients—A 21-year retrospective study. Mycoses.

[4] Patterson T.F., Thompson G.R., Denning D.W., Fishman J.A., Hadley S., Herbrecht R., Kontoyiannis D.P., Marr K.A., Morrison V.A., Nguyen M.H. (2016). Practice Guidelines for the Diagnosis and Management of Aspergillosis: 2016 Update by the Infectious Diseases Society of America. Clin. Infect. Dis..

[5] Donnelly J.P., Chen S.C., Kauffman C.A., Steinbach W.J., Baddley J.W., Verweij P.E., Clancy C.J., Wingard J.R., Lockhart S.R., Groll A.H. (2020). Revision and update of the consensus definitions of invasive fungal disease from the European Organization for Research and Treatment of Cancer and the Mycoses Study Group Education and Research Consortium. Clin. Infect. Dis..

[6] Latgé J.-P., Chamilos G. (2019). *Aspergillus fumigatus* and Aspergillosis in 2019. Clin. Microbiol. Rev..

[7] Segal B.H., Fridlender Z. (2022). Editorial: Neutrophils in Cancer. Front. Immunol..

[8] Verweij P.E., Rijnders B.J.A., Brüggemann R.J., Azoulay E., Bassetti M., Blot S., Calandra T., Clancy C.J., Cornely O.A., Chiller T. (2020). Review of influenza-associated pulmonary aspergillosis in ICU patients and proposal for a case definition: An expert opinion. Intensive Care Med..

[9] European Medicines Agency (2021). Vfend: EPAR–Summary for the Public [Internet] Amsterdam: E.M.A. https://www.ema.europa.eu/en/medicines/human/EPAR/vfend.

[10] Bassetti M., Giacobbe D.R., Agvald-Ohman C., Akova M., Alastruey-Izquierdo A., Arikan-Akdagli S., Azoulay E., Blot S., Cornely O.A., Cuenca-Estrella M. (2024). Invasive fungal diseases in adult patients in intensive care unit (FUNDICU): 2024 consensus definitions from ESGCIP, EFISG, ESICM, ECMM, MSGERC, ISAC, and ISHAM. Intensive Care Med..

[11] Bassetti M., Giacobbe D.R., Vena A., Wolff M. (2021). Antifungal therapy in elderly patients. J. Antimicrob. Chemother..

[12] Nuh A., Ramadan N., Shah A., Armstrong-James D. (2022). Sputum galactomannan has utility in the diagnosis of chronic pulmonary aspergillosis. J. Fungi.

[13] Hamed K., Engelhardt M., Kovanda L.L., Huang J.J., Yan J., Aram J.A. (2023). Post-hoc analysis of the safety and efficacy of isavuconazole in older patients with invasive fungal disease from the VITAL and SECURE studies. Sci. Rep..

[14] Bassetti M., Peghin M., Vena A. (2018). Challenges and solution of invasive aspergillosis in non-neutropenic patients: A review. Infect. Dis. Ther..

[15] Gong Y., Li C., Wang C., Li J., Ding M., Chen D., Lao M. (2020). Epidemiology and mortality-associated factors of invasive fungal disease in elderly patients: A 20-year retrospective study from Southern China. Infect Drug Resist..

[16] Shekhova E., Salazar F., Da Silva Dantas A., Chakraborty T., Wooding E.L., White P.L., Warris A. (2024). Age difference of patients with and without invasive aspergillosis: A systematic review and meta-analysis. BMC Infect Dis..

[17] Chai L.Y., Naesens R., Khoo A.L., Abeele M.V., van Renterghem K., Cartuyvels R., Netea M.G., Gyssens I.C. (2011). Invasive fungal infection in an elderly patient with defective inflammatory macrophage function. Clin Microbiol Infect..

[18] Lao M., Zhang K., Zhang M., Wang Q., Li J., Su L., Ding M., He W., Gong Y. (2020). Clinical features and co-infections in invasive pulmonary aspergillosis in elderly patients. Infect. Drug Resist..

[19] Koehler P., Stecher M., Cornely O.A., Koehler D., Vehreschild J.J., Bohlius J. (2021). Aspergillosis in immunocompromised patients: A comprehensive review. Lancet Infect. Dis..

[20] Bretagne S., Sitbon K., Desnos-Ollivier M., Garcia-Hermoso D., Letscher-Bru V., Cassaing S., Millon L., Morio F., Gangneux J.-P., Hasseine L. (2022). Active surveillance program to increase awareness on invasive fungal diseases: The French RESSIF Network (2012 to 2018). mBio.

[21] Bellmann R., Smuszkiewicz P. (2017). Pharmacokinetics of antifungal drugs: Practical implications for optimized treatment of patients. Infection..

[22] Mellinghoff S.C., Panse J., Alakel N., Behre G., Buchheidt D., Christopeit M., Hasenkamp J., Kiehl M., Koldehoff M., Krause S.W. (2018). Primary prophylaxis of invasive fungal infections in patients with haematological malignancies: 2017 update of the recommendations of the Infectious Diseases Working Party (AGIHO) of the German Society for Haematology and Medical Oncology (DGHO). Ann. Hematol..

[23] Jacobs F., Selleslag D., Aoun M., Sonet A., Gadisseur A. (2012). An observational efficacy and safety analysis of the treatment of acute invasive aspergillosis using voriconazole. Eur. J. Clin. Microbiol. Infect. Dis..

[24] Shin D.H., Yoo S.-J., Jun K.I., Kim H., Kang C.K., Song K.-H., Choe P.G., Park W.B., Bang J.-H., Kim E.S. (2020). Short course of voriconazole therapy as a risk factor for relapse of invasive pulmonary aspergillosis. Sci. Rep..

[25] Bongomin F., Harris C., Hayes G., Kosmidis C., Denning D.W. (2018). Twelve-month clinical outcomes of 206 patients with chronic pulmonary aspergillosis. PLoS ONE.

[26] Maertens J.A., Raad I.I., Marr K.A., Patterson T.F., Kontoyiannis D.P., Cornely O.A., Bow E.J., Rahav G., Neofytos D., Aoun M. (2016). Isavuconazole versus voriconazole for primary treatment of invasive mould disease caused by *Aspergillus* and other filamentous fungi (SECURE): A phase 3, randomised-controlled, non-inferiority trial. Lancet.

[27] Hoenigl M., Jenks J.D., Meis J.F. (2020). Diagnostic and therapeutic challenges of invasive fungal infections in older adults. Aging Res. Rev..

[28] Rocchi S., Sewell T.R., Valot B., Godeau C., Laboissiere A., Millon L., Fisher M.C. (2021). Molecular epidemiology of azole-resistant *Aspergillus fumigatus* in France shows patient and healthcare links to environmentally occurring genotypes. Front. Cell Infect Microbiol..

[29] García-Vidal C., Upton A., Martín-Rabadán P., Meije Y. (2019). Nosocomial infections in elderly patients: Prevention strategies. Clin. Infect. Dis..

[30] Ramos-Ramírez K.E., Soto A. (2020). Sarcopenia, mortalidad intrahospitalaria y estancia hospitalaria prolongada en adultos mayores internados en un hospital de referencia peruano. Acta Med. Peru..

